# Viral Hepatitis in Korea: Past, Present, and Future

**DOI:** 10.5005/jp-journals-10018-1170

**Published:** 2016-07-09

**Authors:** Hyo-Suk Lee

**Affiliations:** Liver Center, Myongji Hospital, Goyang-si, Gyeonggi-do, Korea

**Keywords:** Blood screening, Epidemiology, Hepatitis B, Hepatitis C, Hepatocellular carcinoma, Korea, Vaccination.

## Abstract

Korea has been one of the endemic areas of hepatitis B virus (HBV; exclusively genotype C) infection since ancient times. The epidemiology of HBV in Korea is easily expected to have changed over the last two decades owing to the high coverage rate of universal HBV vaccination. The main mode of hepatitis C virus (HCV) infection may have been effectively blocked since 1992 when compulsory blood screening for anti-HCV was implemented, and consequently, the prevalence of anti-HCV is also expected to have markedly decreased during the last two decades. This review will briefly describe what really happened during the last couple of decades in the epidemiology of HBV and HCV and in the incidence and mortality rates of liver cirrhosis (hepatic failure) and hepatocellular carcinoma in Korea.

**How to cite this article:**

Lee H-S. Viral Hepatitis in Korea: Past, Present, and Future. Euroasian J Hepato-Gastroenterol 2016;6(1):62-64.

## INTRODUCTION

Korea has been one of the endemic areas of hepatitis B virus (HBV; exclusively genotype C) infection since ancient times. A comparison of ancient HBV DNA sequences obtained from an HBV-infected 16^th^-century mummified child excavated in Yangju, Korea, with contemporary HBV DNA suggests that the origin of Korean HBV sequence dates back to at least 3,000 years.^1^ These findings are in line with Dr Mizokami’s group’s estimates that human HBV DNA existed in East Asia approximately 3,000 years ago or earlier.

The epidemiology of HBV in Korea is easily expected to have changed over the last two decades owing to the high coverage rate of universal HBV vaccination, which resulted in dramatic reductions in the prevalence of HBsAg and the incidence of chronic hepatitis, cirrhosis, hepatic failure, and subsequent hepatocellular carcinoma (HCC). This might be further accelerated by the recent progress in the antiviral therapy of chronic hepatitis B. In contrast, nationwide attention and effort were relatively less to HCV epidemiology than to HBV epidemiology. The main mode of HCV infection may have been effectively blocked since 1992 when compulsory blood screening for anti-HCV was implemented, and consequently, the prevalence of anti-HCV is also expected to have markedly decreased during the last two decades along with the aging cohorts of patients with chronic HCV infection. This review will briefly describe what really happened during the last couple of decades in the epidemiology of HBV and HCV and in the incidence and mortality rates of liver cirrhosis (hepatic failure) and HCC in Korea.

## IMPACT OF HBV VACCINATION AND COMPULSORY BLOOD SCREENING FOR ANTI-HCV

The HBV vaccination was recommended for all newborns in 1983 (coverage rate: 79.7%), and was then integrated into the Expanded Program on Immunization (EPI) in 1995 (coverage rate: 98.9%).^2^ Therefore, the mother-to-infant perinatal transmission, which was the most important mode of infection, as well as horizontal transmission in childhood was effectively prevented; therefore, the prevalence of HBsAg has markedly decreased in young age groups, especially in teens, from 14.2 to 0.1%, during the last 30 years, and consequently, the prevalence of HBsAg in the general population also decreased from 8.6% in 1982 to 3.0% in 2011.^3^ However, the prevalence in those in their 50s was still as high as 5% in 2011. On the contrary, the prevalence of anti-HCV, owing to the compulsory blood screening for anti-HCV since 1992 and to the fact that HCV-infected adults are getting older, markedly decreased in elderly people over 60 years of age from 5.5% in 1990–1994 to 1.3% in 2009,^4^ and also decreased accordingly from 1.7 to 0.78% in the general population. In other words, the prevalence of HBsAg at aged with the highest incidence of HCC (those in their 50s) still remains high (5%); in contrast, the prevalence of anti-HCV at aged with the highest incidence of HCC (those in their 60s) markedly decreased (5.5–0.78%), suggesting that the incidence of HCV-related HCC has been more rapidly decreasing than HBV-related HCC. Actually, the data obtained from 5,024 HCC patients at Seoul National University Hospital (SNUH) showed that the relative etiologic role of HCV among virus-associated HCCs decreased from 10.6% in 1991–2000 to 4.2% in 2001–2010 (unpublished data). However, the official report of Statistics Korea (2012)^5^ showed that despite the fact that HCV-related HCC has been decreasing, there was virtually no change in the mortality rates (approximately 25/100,000) of HCC in the 10 years since 2002, suggesting that HCC due to reasons other than viral origin (HBV and/or HCV) such as nonalcoholic steatohepatitis (NASH) has recently been increasing, and it was actually confirmed that HCC of nonviral origin has been increasing since the past 10 years.^6^

## IMPACT OF ANTIVIRAL THERAPY ON THE PROGNOSIS OF HCC

Although the sustained virological response (SVR) rates to peg-interferon/ribavirin combination therapy is higher in Korean patients than those in Western countries, the influence of HCV antiviral therapy on the incidence of HCC may not be substantial because only a small proportion (8.6%) of chronic HCV-infected subjects took antiviral treatment (unpublished data). On the contrary, most patients with chronic hepatitis B took oral antiviral therapy, which may impact the incidence and the survival of liver cirrhosis and HCC patients. A study done at SNUH revealed that the median survivals of HCC patients were significantly longer in entecavir-treated than in naïve patients (unpublished data). The prolonged survival was thought to be due to the effective HBV antiviral therapy on the prevention of hepatic failure without HCC progression rather than the prevention of recurrence or occurrence of HCC. Although antiviral therapy may decrease the incidence of HBV-associated HCC, it is well known that it cannot totally eliminate the risk of developing HCC; it might rather increase the chance of developing HCC owing to the prolongation of survival time of HBV carriers. According to data from Statistics Korea (2012),^5^ the mortality rates due to hepatic failure continuously decreased during the last 10 years, while those due to HCC progression remained stationary, which might be also explained by the increment of the risk population for HCC due to increased survival time of chronic HBV carriers with antiviral therapy in addi-tion to the increment of HCC of nonviral origin such as NASH.

## PERSPECTIVES

Most HCV carriers in Korea are elderly people, older than 60; the prevalence of anti-HCV is surely anticipated to continuously decrease as they are getting older (“cohort effect”), and furthermore, the availability of effective oral direct antiviral agent (DAA) against HCV is expected to accelerate the eradication of virtually all of HCV infection in Korea within a decade followed by the elimination of HCV-related HCC in a couple of decades. In contrast, despite the launch of an effective hepatitis B vaccination program more than 20 years ago, HBV infection still remains a disease of significant health burden in Korea. The number of HBV carriers in people with the highest risk of developing HCC will remain rather stationary; the prevalence of HBsAg in those in their 50s, when HCC occurs at its peak, is expected to decrease only by 1% from 5 to 4% in the next 20 years, suggesting that HBV-associated HCC will continue to develop during the next 20 years.

In summary, hepatitis B vaccination and compulsory blood screening for HCV markedly decreased the prevalence of HBsAg and anti-HCV in children and the elderly respectively. Consequently, the relative etiologic role of HCV over HBV has rapidly decreased during the last 20 years. HBV–HCC will continue to develop in the next two decades because the prevalence of HBsAg in older age groups over 50 years of age remains stationary or, at best, slightly decreases. An effective antiviral therapy against HBV causes marked reduction in mortality due to hepatic failure but not substantially due to HCC itself. Owing to the availability of effective oral DAAs against HCV and to the fact that elderly anti-HCV-positive subjects are getting older and there is negligible number of new HCV-infected cases, HCV infection is expected to be virtually eradicated in a couple of decades. Among nonviral liver diseases, alcoholic liver disease remains an extremely neglected public health problem with no prospects for imminent improvement. NASH is likely to continuously increase to surpass most of the viral liver diseases in the next three decades in Korea.

## References

[1] Kahila Bar-Gal G, Kim MJ, Klein A, Shin DH, Oh CS, Kim JW, Kim TH, Kim SB, Grant PR, Pappo O, et al. (2012). Tracing hepatitis B virus to the 16th century in a Korean mummy.. Hepatology.

[2] Park NH, Chung YH, Lee HS (2010). Impacts of vaccination on hepatitis B viral infections in Korea over a 25-year period.. Intervirology.

[3] Ministry of Health and Welfare, 2012 [accessed 2013].. http://stat.mw.go.kr/front/statDB/statDBView.jsp?menuId=11&sttsDataSeq=100.

[4] Kim do Y, Kim IH, Jeong SH, Cho YK, Lee JH, Jin YJ, Lee D, Suh DJ, Han KH, Park NH, et al. (2013). A nationwide seroepidemiology of hepatitis C virus infection in South Korea.. Liver Int.

[5] Statistics Korea, 2012 (e-나라지표).. http://www.index.go.kr/portal/main/EachDtlPageDetail.do?idx_cd=1012.

[6] Cho EJ, Kwack MS, Jang ES, You SJ, Lee JH, Kim YJ, Yoon JH, Lee HS (2011). Relative etiological role of prior hepatitis B virus infection and nonalcoholic fatty liver disease in the development of non-B non-C hepatocellular carcinoma in a hepatitis B-endemic area.. Digestion.

